# Acquired hypothalamic obesity as α-MSH deficiency syndrome: a paradigm shift with therapeutic impact

**DOI:** 10.1186/s40348-026-00260-1

**Published:** 2026-10-15

**Authors:** Martin Wabitsch, Kim Ulrich, Christian Denzer

**Affiliations:** 1 Division of Pediatric Endocrinology and Diabetes, Department of Pediatrics and Adolescent Medicine, Ulm University Medical Center, Ulm, Germany; 2 German Center for Child and Adolescent Health (DZKJ), partner site Ulm, Ulm, Germany

## Abstract

Recent findings by Miller et al. (https://doi.org/10.1056/NEJMoa2512275) suggest a fundamental shift in the understanding of acquired hypothalamic obesity (aHO), proposing that this condition should be viewed as an α-melanocyte-stimulating hormone (α-MSH) deficiency syndrome. Building on these observations, we discuss the proposed underlying pathophysiological mechanisms and argue that aHO can be conceptualized as an injury-induced loss of melanocortin tone, analogous to an α-MSH deficiency. The marked clinical response to setmelanotide in affected individuals indicates that downstream melanocortin-4 receptor (MC4R)-dependent pathways remain functionally intact, supporting the concept of a hormone deficiency–like state. We further explore the implications of this paradigm change, highlighting how this new framework may fundamentally change the diagnosis and management of patients with aHO.

## Main text

Acquired hypothalamic obesity (aHO) is a devastating complication of hypothalamic injury—most commonly after craniopharyngioma or its treatment—characterized by rapid, intractable weight gain, hyperphagia and a disproportionate reduction in energy expenditure [1]. Despite decades of study, effective, mechanism-based therapies have been lacking. Here, we expand on the proposed reframing of aHO as an injury-induced state of reduced melanocortin tone - functionally akin to α-melanocyte-stimulating hormone (α-MSH) deficiency at the level of central melanocortin-4-receptor (MC4R) circuits. This is in line with findings from a recent phase 3 clinical trial [2], providing a rationale for the emerging therapeutic efficacy of setmelanotide, a MC4R agonist, as a form of hormone replacement therapy in aHO.

The hypothalamus acts as the central regulator of appetite and energy balance, integrating peripheral and central signals through its various nuclei, including the arcuate nucleus (ARC) and paraventricular nucleus (PVN). Traditionally, aHO has been explained by structural damage causing disruption of these centers, leading to impaired satiety signaling, disinhibited vagal tone, and consequent metabolic derangements [3, 4]. However, this “damage to the satiety center”- model may be incomplete.

Recent evidence supports a model of reduced melanocortin tone. α-MSH is derived from the precursor pro-opiomelanocortin (POMC), predominantly expressed in hypothalamic ARC neurons. These neurons produce α-MSH, which acts via MC4R expressed widely in the central nervous system —particularly in the PVN, dorsal motor nucleus of the vagus (DMV), nucleus of the solitary tract (NTS)—and peripherally, including vagal afferents and efferents and enteroendocrine cells [5, 6]. The binding of α-MSH to MC4R triggers anorexigenic pathways reducing food intake and increasing energy expenditure.

Crucially, studies have shown that hypothalamic injury may disrupt POMC neuron function and α-MSH production. Consistent with this view, clinical and translational work suggests that hypothalamic lesions can dysregulate leptin–POMC–melanocortin signaling and thereby reduce endogenous melanocortin drive to MC4R pathways [7]. The efficacy of setmelanotide in reducing weight and hyperphagia in patients with aHO in the phase 3 clinical trial [2] further implies that the MC4R pathways remain sufficiently intact and responsive, enabling meaningful weight loss especially within the first 6 months of treatment, despite impaired upstream signaling [2]. These observations raise the possibility of a partial “set-point”-shift towards pre-injury weight regulation, which - if sustained - could potentially support longer-term weight control [3]. However, rather than directly quantifying endogenous α-MSH deficiency, the therapeutic response demonstrates that MC4R-dependent signaling remains pharmacologically accessible and that MC4R agonism can compensate for disrupted melanocortin tone.

**Fig. 1:**
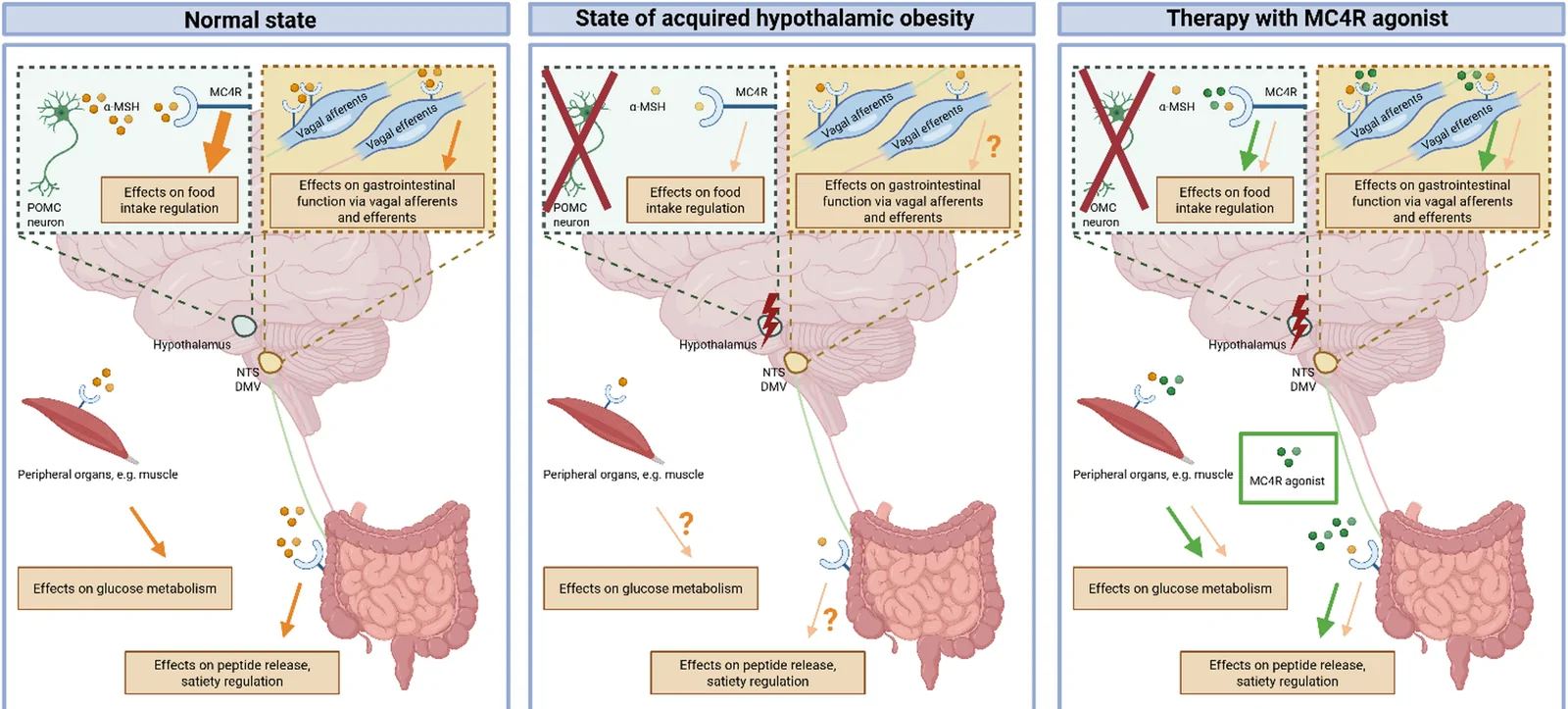
Acquired hypothalamic obesity as a hormone deficiency syndrome. The left panel illustrates the proposed physiological role of α-MSH in central and peripheral systems. The middle panel depicts the hypothesized central α-MSH deficiency in acquired hypothalamic obesity, primarily resulting from damage to POMC neurons in the hypothalamus, thereby hypothetically reducing α-MSH signaling. A reduction in peripheral α-MSH might appear in patients with additional pituitary or hypothalamic-pituitary injury, as the pituitary has been proposed as a potential source of circulating α-MSH. However, this peripheral relationship remains hypothetical. The right panel presents a model showing how therapy with an MC4R agonist may increase downstream MC4R signaling at central and selected peripheral sites, thereby mitigating some consequences of impaired upstream melanocortin signaling

This shift in understanding moves the focus from nonspecific damage to satiety centers to damage to the hormone-producing neurons themselves. It suggests reframing aHO as a hormonal deficiency syndrome akin to classical endocrinopathies. The conceptual analogy is compelling: just as hypothyroidism arises from thyroid hormone deficiency treatable by replacement, aHO may stem from α-MSH deficiency treatable by MC4R agonists like setmelanotide (Fig. 1).

The anatomical and functional findings are consistent with this model. POMC neurons are concentrated in the hypothalamic ARC but are also present in the NTS [5]. Lesion or ablation of ARC POMC neurons leads to obesity, increased fat mass and glucose intolerance, unlike ablation of NTS POMC neurons [5]. Moreover, it appears that only ARC POMC neurons respond directly to leptin and mediate downstream effects such as α-MSH production. Thus, when this signaling is disrupted due to structural damage in aHO, this likely contributes to an α-MSH deficiency [8]. Meanwhile, MC4R expression spans hypothalamic nuclei like the PVN, hindbrain centers including DMV and NTS, and extends to the cortex, thalamus, brainstem, spinal cord, vagal afferents and enteroendocrine cells, linking central and peripheral control of appetite and metabolism [5, 6, 9].

Melanocortin agonists delivered to the DMV or NTS reduce food intake and gastric motility, highlighting the hormone’s multifaceted role [6]. Moreover, MC4R activation has been shown to stimulate the release of peptide YY (PYY) from L-cells in human colon tissue, which also co-express glucagon-like peptide-1 (GLP-1) [6], further promoting satiety. Furthermore, systemic α-MSH, released from the pituitary gland, acts on a variety of peripheral tissues [10]. Thus, defective MC4R activation may not only disrupt central satiety perception but also peripheral digestive and metabolic signaling, contributing to the complexity of aHO. The extent to which these peripheral mechanisms contribute to human aHO remains uncertain.

The clinical implications of this paradigm shift are profound. Until recently, treatments for aHO were largely ineffective, focusing on symptomatic management rather than the underlying cause. In 2026, the FDA and European Commission expanded their approval of setmelanotide to include aHO, making it the first approved therapy specifically indicated for aHO in adults and children aged 4 years and older. This specific therapeutic “replacement” approach may transform management by addressing the primary pathophysiology and restoring melanocortin signaling downstream of the suggested α-MSH deficiency.

The proposed model also raises the question of whether the anatomical distribution and severity of hypothalamic injury may influence the therapeutic response to setmelanotide. Patients with aHO show heterogeneity in both lesion localization and extent [4, 11]. In some patients, damage may predominantly disrupt upstream POMC-related pathways, whereas more extensive lesions may additionally involve downstream α-MSH-responsive circuits. Notably, posterior hypothalamic involvement and greater lesion severity are associated with an increased risk and magnitude of obesity [4].

Future research should therefore assess whether clinical, biochemical, and imaging characteristics of hypothalamic injury can predict the response to MC4R agonism. This is particularly relevant because no established biomarkers are currently available to identify patients who are most likely to respond to setmelanotide. Structural imaging characteristics, post-injury BMI trajectories, hyperphagia severity, and early treatment response, considered individually or in combination, may support a more personalized therapeutic approach and facilitate the selection of patients most likely to benefit. Ultimately, biomarkers reflecting central and peripheral melanocortin pathway integrity could help guide patient selection and monitor treatment response.

In conclusion, we recognize a paradigm shift conceptualizing aHO as an injury-induced state of reduced melanocortin tone -functionally resembling α-MSH deficiency - arising from disruption of POMC neuron output. The marked efficacy of setmelanotide provides strong functional evidence for a central role of the melanocortin pathway in aHO and the hypothesis that MC4R-dependent signaling remains pharmacologically accessible and that MC4R agonism can compensate, at least in part, for impaired upstream melanocortin signaling. This is consistent with the concept of a neuroendocrine deficiency syndrome and further supports a shift from largely symptomatic management toward mechanism-based therapy in this challenging disorder.

## Data Availability

No datasets were generated or analysed during the current study.
